# Improved flower pollination algorithm for identifying essential proteins

**DOI:** 10.1186/s12918-018-0573-y

**Published:** 2018-04-24

**Authors:** Xiujuan Lei, Ming Fang, Fang-Xiang Wu, Luonan Chen

**Affiliations:** 1 School of Computer Science, Shaanxi Normal University, Xi’an, 710119 China; 2 Division of Biomedical Engineering and Department of Mechanical Engineering, University of Saskatchewan, Saskatoon, Canada; 3 Key Laboratory of Systems Biology, CAS center for Excellence in Molecular Cell Science, Innovation Center for Cell Signaling Network, Institute of Biochemistry and Cell Biology, Shanghai Institutes for Biological Sciences, Chinese Academy of Sciences, Shanghai, 200031 China

## Abstract

**Background:**

Essential proteins are necessary for the survival and development of cells. The identification of essential proteins can help to understand the minimal requirements for cellular life and it also plays an important role in the disease genes study and drug design. With the development of high-throughput techniques, a large amount of protein-protein interactions data is available to predict essential proteins at the network level. Hitherto, even though a number of essential protein discovery methods have been proposed, the prediction precision still needs to be improved.

**Methods:**

In this paper, we propose a new algorithm, improved Flower Pollination algorithm (FPA) for identifying Essential proteins, named FPE. Different from other existing essential protein discovery methods, we apply FPA which is a new intelligent algorithm imitating pollination behavior of flowering plants in nature to identify essential proteins. Analogous to flower pollination is to find optimal reproduction from the perspective of biological evolution, and the identification of essential proteins is to discover a candidate essential protein set by analyzing the corresponding relationships between FPA algorithm and the prediction of essential proteins, and redefining the positions of flowers and specific pollination process. Moreover, it has been proved that the integration of biological and topological properties can get improved precision for identifying essential proteins. Consequently, we develop a *GSC* measurement in order to judge the essentiality of proteins, which takes into account not only the Gene expression data, Subcellular localization and protein Complexes information, but also the network topology.

**Results:**

The experimental results show that FPE performs better than the state-of-the-art methods (DC, SC, IC, EC, LAC, NC, PeC, WDC, UDoNC and SON) in terms of the prediction precision, precision-recall curve and jackknife curve for identifying essential proteins and also has high stability.

**Conclusions:**

We confirm that FPE can be used to effectively identify essential proteins by the use of nature-inspired algorithm FPA and the combination of network topology with gene expression data, subcellular localization and protein complexes information. The experimental results have shown the superiority of FPE for the prediction of essential proteins.

## Background

Essential proteins are indispensable in the cellular life for the survival or development of an organism. Even though the deletion of only one of these proteins will cause a lethal flaw on an organism [1]. Studies have shown that essential proteins are related to disease genes [2] and contribute to the prediction of drug targets [3]. Therefore, identifying essential proteins is not only conducive to the understanding of minimal requirements for cellular life, but also important for the study of disease genes [4].

The traditional methods of identifying essential proteins are biological experiments, such as gene knockouts [5], RNA interference [6] and conditional knockouts [7], these biological experiment discovery methods are accurate, but time-consuming, low efficiency and expensive. Up to now, many computational methods for predicting essential proteins have been proposed. Particularly with the rapid development of high-throughput technologies, such as yeast two-hybrid screens [8], tandem affinity purification [9] and mass spectrometric analysis [10], a large amount of protein interaction data is detected, which provide new possibilities for the identification of essential proteins. It is becoming increasingly important to predict essential proteins by computational methods based on protein interaction data.

The identification of essential proteins based on protein-protein interaction (PPI) networks by using various topological properties is a very hot topic. Until now, many essential protein discovery methods have been proposed, while most of these methods are based on proteins with highly connected neighbors tend to be essential, named the “centrality-lethality” rule [11], such as Degree Centrality (DC) [11], Betweenness Centrality (BC) [12, 13], Closeness Centrality (CC) [14], Subgraph Centrality (SC) [15], Eigenvector Centrality (EC) [16], Information Centrality (IC) [17]. Moreover, there are also two neighborhood-based methods: Neighborhood Centrality (NC) [18] and Local Average Connectivity-based method (LAC) [19].

The above methods depend on the PPI networks to identify essential proteins and have made great progresses in the essential protein discovery tasks. However, it is still a challenge to improve the prediction precision owing to the PPI networks obtained by high-throughput technologies contain many false positives which may greatly affect the precision of identification of essential proteins [20]. Furthermore, these methods neglect the inherent biological significance of essential proteins. Hence, to reduce the effect of noise in the PPI networks, the researchers have tried to achieve higher precision of identifying essential proteins by integrating other biological information. For example, ION [21] used the orthologous information with the PPI networks. A method PeC [22] integrated gene expressions and PPI networks, Peng et al. [23] proposed UDoNC by integrating domains and PPI networks. Zhong et al. [24] used a feature selection method by collecting 26 different biological and topological features to identify essential proteins, SON [25] integrated subcellular localization, orthology and PPI networks, United complex Centrality (UC) [26] utilized protein complexes information to predict essential proteins. Besides the methods mentioned above, some researchers integrated topological or biological information to construct dynamic networks. For example, Xiao et al. [27] constructed an active PPI network to predict essential proteins.

Flower pollination algorithm (FPA) [28] is a nature-inspired intelligent optimization algorithm that considers the characteristics of flower pollination, which proposed by Yang in 2012. There are two main patterns of the pollination process viz. abiotic pollination and biotic pollination. Pollinators can be biotic or abiotic depending on the type of pollination. For biotic pollination, pollinators are some animals such as insects and birds. This type of pollination is called as global pollination. About 90% of the pollination is biotic in nature. For abiotic pollination, pollinators are natural resources such as wind, water and soil. This type of pollination is local pollination. The global pollination and local pollination are two main steps of FPA, these two steps are regulated by the switch probability. FPA has been applied in various practical problems such as clustering [29], feature selection [30] and multi-objective optimization problem [31]. Consequently, the efficiency of FPA makes it possible for addressing the problem of predicting essential proteins.

In this study, we develop a new algorithm, named FPE, based on improved FPA to identify essential proteins by integrating gene expression data, subcellular localization and protein complexes information with the topological properties of PPI networks. Different from other essential protein discovery methods that already exist, we take advantage of the improved version of FPA to provide a new perspective for the identification of essential proteins. Also, our algorithm FPE integrates biological properties and topological properties of PPI networks to assess the essentiality of proteins and further improve the performance of prediction results. In order to evaluate the effectiveness of the proposed algorithm FPE, we apply it to the PPI networks and compare with ten previous essential protein discovery methods: DC [11], SC [15], IC [17], EC [16], LAC [19], NC [18], PeC [22], WDC [32], UDoNC [23] and SON [25]. The experimental results on the identification of yeast essential proteins show that FPE outperforms the ten previously proposed methods in terms of the prediction precision, as well as the precision-recall curve and the jackknife curve. The modularity of proteins and the effect of the parameter *p* on the prediction results is also discussed.

The rest of this paper is organized as follows. We first introduce the basic knowledge of FPA. Then we present how to combine FPA with the identification of essential proteins, as well as the measurement for evaluating the essentiality of proteins. Next, the performance of FPE is validated by using a series of comparison experiments and the analysis of experimental results are also described. We conclude this study at the end.

## Methods

The FPE algorithm is used to identify essential proteins on the basis of the combination of improved flower pollination algorithm with the gene expression data, subcellular localization and protein complexes information.

### Flower pollination algorithm (FPA)

FPA [28] is a population-based global optimization technique, which is motivated by the pollination process of flowers. Pollination can be divided into two types, i.e., self-pollination and cross-pollination. Self-pollination takes place between the flowers of the same plant species while cross-pollination can occur from the flowers of different plant species. Biotic pollinators such as insects and birds can fly long distances causing cross-pollination, which thus can be considered as global pollination. Abiotic pollinators are the natural resources such as wind and water that are unable to take away pollens to long distances causing self-pollination, which is local pollination. The local pollination and global pollination interchange is controlled by a parameter *p* ∈ [0, 1] defined by so called switch probability. Table 1 shows the basic knowledge of FPA.

**Table 1:** The basic knowledge of FPA

	Local pollination	Global pollination
Types	Self-pollination (Abiotic)	Cross-pollination (Biotic)
Flowers	Same plant species	Different plant species
Pollinators	Wind, water	Insects, birds

In FPA, it is assumed that each plant only has one flower and each flower only has one pollen gamete for simplicity. Therefore, a flower or pollen gamete represented by a position vector that denotes as a candidate solution of the optimization problem. Flower pollens will be transferred in global pollination and local pollination.

In the global pollination, pollens are carried to long distances by pollinators, such as insects, because these pollinators can fly and move in a longer distance.

1 $$ {x}_i^{t+1}={x}_i^t+F\left({x}_i^t- gbest\right) $$

where ${x}_i^t$ is the pollen *i* at iteration *t*, and *gbest* is the current best solution which is found among all solutions at the current iteration. The parameter *F* is the strength of the pollination, namely a step size, we use a Lévy flight to represent that insects move over a long distance with various distance steps. That is, *F* > 0 and follows Lévy distribution:

2 $$ F\sim \frac{\lambda \varGamma \left(\lambda \right)\sin \left(\pi \lambda /2\right)}{\pi}\frac{1}{s^{1+\lambda }},\left(s\gg {s}_0>0\right) $$

where *Γ*(*λ*) is the standard gamma function, and this distribution is valid for large steps s > 0.

The local pollination occurs within a limited range thanks to pollinators like wind or water, which can be defined as:

3 $$ {x}_i^{t+1}={x}_i^t+\varphi \left({x}_j^t-{x}_k^t\right) $$

where ${x}_j^t$ and ${x}_k^t$ are pollen from the different flowers of the same plant species. This substantially models the flower constancy in a limited neighborhood. Mathematically, if ${x}_j^t$ and ${x}_k^t$ come from the same plant species or select from the same population, this can be a local random walk if *φ* follows the uniform distribution in [0, 1].

From the biological evolution point of view, it is a fact that the aim of the flower pollination is achieving the optimal reproduction of the flowering plants. The pollinator’s movement towards the optimal solution is represented by the global optimum found by FPA, namely, the most suitable reproduction and pollination are found, which is represented by *gbest*.

Taking the basic principle of FPA algorithm into consideration, we design a new FPE algorithm, which is an improved version of FPA algorithm to identify essential proteins. In the FPE algorithm, the position of a pollen is represented as a set of candidate essential proteins contained *Q* proteins.

### Improved flower pollination algorithm for essential proteins identification (FPE)

In this section, we will use an improved FPA algorithm to develop a new algorithm, named FPE. Table 2 illustrates the corresponding relationships between FPA algorithm and the identification of essential proteins.

**Table 2:** The corresponding relationships between FPA algorithm and the identification of essential proteins

FPA algorithm	The identification of essential proteins
Pollen	A candidate essential protein set
Pollen’s position	The serial numbers of *Q* candidate essential proteins
Fitness function	The measurement of proteins’ essentiality
Pollination process	The process of identifying essential proteins

#### Pollen’s position

A PPI network is described by an undirected graph *G* (*V, E*), where *V* denotes a set of nodes that are proteins and *E* denotes a set of edges of PPI network.

In the basic FPA, the position of a flower is viewed as a candidate solution for the optimization problem that needs to be solved. Nevertheless, in our FPE algorithm, the positions of flowers are redefined as the candidate sets of essential proteins and each candidate set consists of *Q* proteins. A pollen can be encoded as a candidate essential protein set *H* = {*h*_*1*_, *h*_*2*_,…*h*_*Q*_}, where each of these elements represents the serial number of a protein.

#### Pollination process

We redesign the formulas for updating the pollen’s positions considering that the basic FPA algorithm is continuous form while our proposed algorithm is discrete form.

In the global pollination of FPA algorithm represented by formula (1), on the one hand, pollen constantly move to the global optimal solution, on the other hand, Lévy distribution is used to make the pollen move in a longer distance.

Inspired by the global pollination of basic FPA, we consider its two aspects mentioned above in a comprehensive way to update the position of pollen, hence, in the global pollination of our FPE algorithm, the position of pollen is defined as follows:

4 $$ {L}_i^{t+1}= cat\left(\mathit{\dim},\kern0.5em {L}_i^{t^{\prime }},\kern0.5em RANDOM\right) $$

where the *cat* operation is the function that forms the position vector of a pollen. The value of *dim* is 1, which indicates that two position vectors obtained by ${L}_i^{t^{\prime }}$ and *RANDOM* are concatenated in a column in our FPE algorithm. Then the *RANDOM* indicates that a global search in *V* is performed to update the position of pollen. Finally, the pollen’s new position is obtained by using *cat* function which can connect the position vectors obtained by ${L}_i^{t^{\prime }}$ and *RANDOM* to guarantee that the pollen’s new position ${L}_i^{t+1}$ not only keeps moving towards the global optimal solution, but also searches in a global scope. ${L}_i^{t^{\prime }}$ can be represented as follows:

5 $$ {L}_i^{t^{\prime }}= intersect\left({L}_i^t, Gbest\right) $$

where the *intersect* function denotes that the elements in ${L}_i^t$ intersect with the elements in *Gbest*. Here, the elements in *Gbest* are those of a certain proportion in *Gbest*. This can mimic the update of pollen’s position in the basic FPA algorithm so that the pollen is constantly approaching the global optimum *Gbest*.

In the local pollination, the pollen’s position remains unchanged, which is represented as:

6 $$ {L}_i^{t+1}={L}_i^t $$

**Fig. 1:**
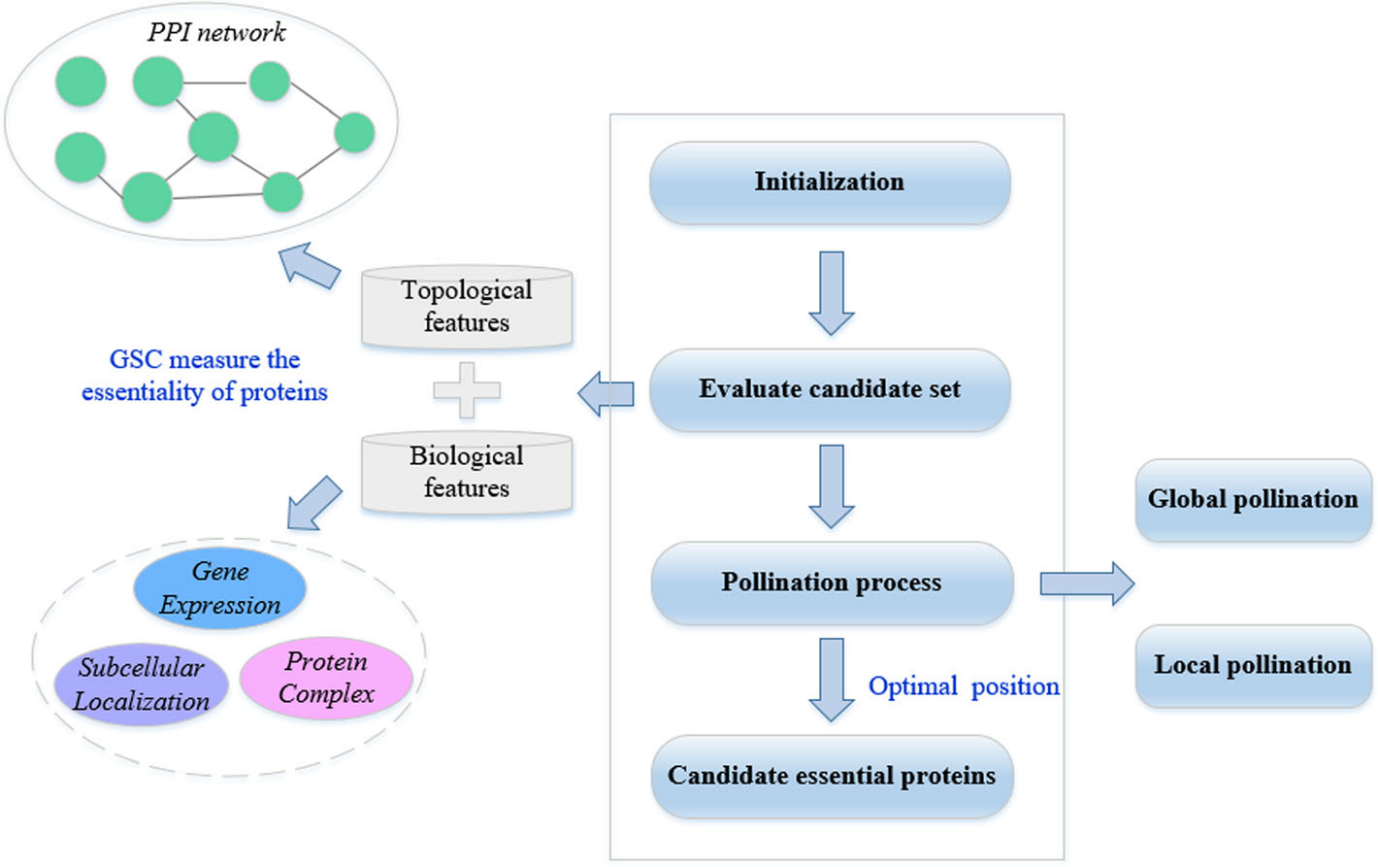
The overall flow of FPE algorithm

The overall flow of the FPE algorithm is shown in Fig. 1.

### The measurement of the essentiality of proteins (GSC)

According to the aforementioned analysis and the conclusions from previous studies, we have known that the position of a flower can be viewed as a candidate solution set of essential proteins and each candidate set consists of *Q* proteins. Then the measurement of the proteins’ essentiality should be needed. Accordingly, we define a new measurement to determine the essentiality of a pollen represented by the *Q* candidate essential proteins, called *GSC*, which consists of three types of information, gene expression data, subcellular localization and protein complexes information. *GSC* can be used to assess the quality of each candidate solution, which corresponds to the fitness function of FPA.

The *GSC* is a measure of combining the centrality measure *PeC*, subcellular localization *SL* and protein complexes *PC*. Subsequently, we will introduce them in detail.

For a candidate set *H* = {*h*_*1*_, *h*_*2*_,…*h*_*Q*_}, where each element *h*_*i*_ denotes a candidate essential protein, its essentiality is evaluated by *GSC*(*H*):

7 $$ GSC(H)=\sum \limits_{i=1}^Q\left\{ SL\times \left[\alpha \times PeC+\left(1-\alpha \right)\times PC\right]\right\} $$

where the parameter *α* is a constant between [0, 1], which is used to adjust the proportions of three types of information. When *α* = 0, only the information about protein complexes and subcellular localization is considered, and when *α* = 1, only the information about subcellular localization and gene expression data with the PPI network is considered. To start with, we use *α* = 0.5 as an initial value and then it has been certified that *α* = 0.6 works better for most applications from our parametric analysis.

Next, we will introduce how to integrate gene expression data, subcellular localization and protein complexes information with the topological properties of PPI networks to determine the proteins’ essentiality.

#### PeC

As we know the edge clustering coefficient (*ECC*) can describe the closeness of two connected nodes in a PPI network. The *ECC* is an important measure to represent the topological properties of PPI networks and it has been proved that *ECC* has a good performance in identifying protein complexes and essential proteins. Furthermore, Pearson correlation coefficient (*PCC*) is a measure that is used to evaluate how likely two interacting proteins are co-expressed. Based on gene expression data and protein-protein interaction data, the centrality method PeC [22] using *ECC* and *PCC* is a very effective essential protein discovery method.

Given a PPI network *G* (*V*, *E*), where a node *i* ∈ *V* denotes a protein and an edge (*i, j*) ∈*E* connecting node *i* and node *j*, its edge clustering coefficient *ECC* (*i*, *j*) can be defined by the following formula:

8 $$ ECC\left(i,j\right)=\frac{\left|{N}_i\cap {N}_j\right|}{\mathit{\min}\left\{{d}_i,{d}_j\right\}} $$

where *N*_*i*_ and *N*_*j*_ denote the set of all neighbors of protein *i* and *j*, respectively, *d*_*i*_ and *d*_*j*_ denote the degree of protein *i* and *j*, respectively.

*X* = (*x*_*1*_, *x*_*2*_, …, *x*_*n*_) and *Y* = (*y*_*1*_, *y*_*2*_, …, *y*_*n*_) are two sequences of gene expressions, *PCC* is calculated by:

9 $$ PCC\left(i,j\right)=\frac{\sum_{i=1}^T\left({x}_i-\mu (x)\right)\left({y}_i-\mu (y)\right)}{\sqrt{\sum_{i=1}^T{\left({x}_i-\mu (x)\right)}^2\bullet {\sum}_{i=1}^T{\left({y}_i-\mu (y)\right)}^2}} $$

The value of *PCC* is between − 1 and 1. The probability that protein *i* and *j* are co-clustered can be calculated as follows:

10 $$ {p}_c\left(i,j\right)= ECC\left(i,j\right)\times PCC\left(i,j\right) $$

Given a protein *i*, its *PeC*(*i*) is defined as follows:

11 $$ PeC(i)=\sum \limits_{v\in {n}_i}{p}_c\left(i,v\right) $$

where *n*_*i*_ denotes the set of all neighbors of protein *i*. It is obvious that a protein gets higher values of *ECC* and *PCC*, it will obtain a relatively higher value of *PeC* and thus tends to be an essential protein.

#### Subcellular localization

It is well known that subcellular localization is a significant property of essential proteins and a protein must be appeared in an appropriate subcellular location. The basic idea that we use subcellular localization information to identify essential proteins is that essential proteins appear more often in certain subcellular locations [25]. Consequently, we hypothesize that the proteins, which are in the same subcellular location as the known essential proteins are tend to be essential.

**Fig. 2:**
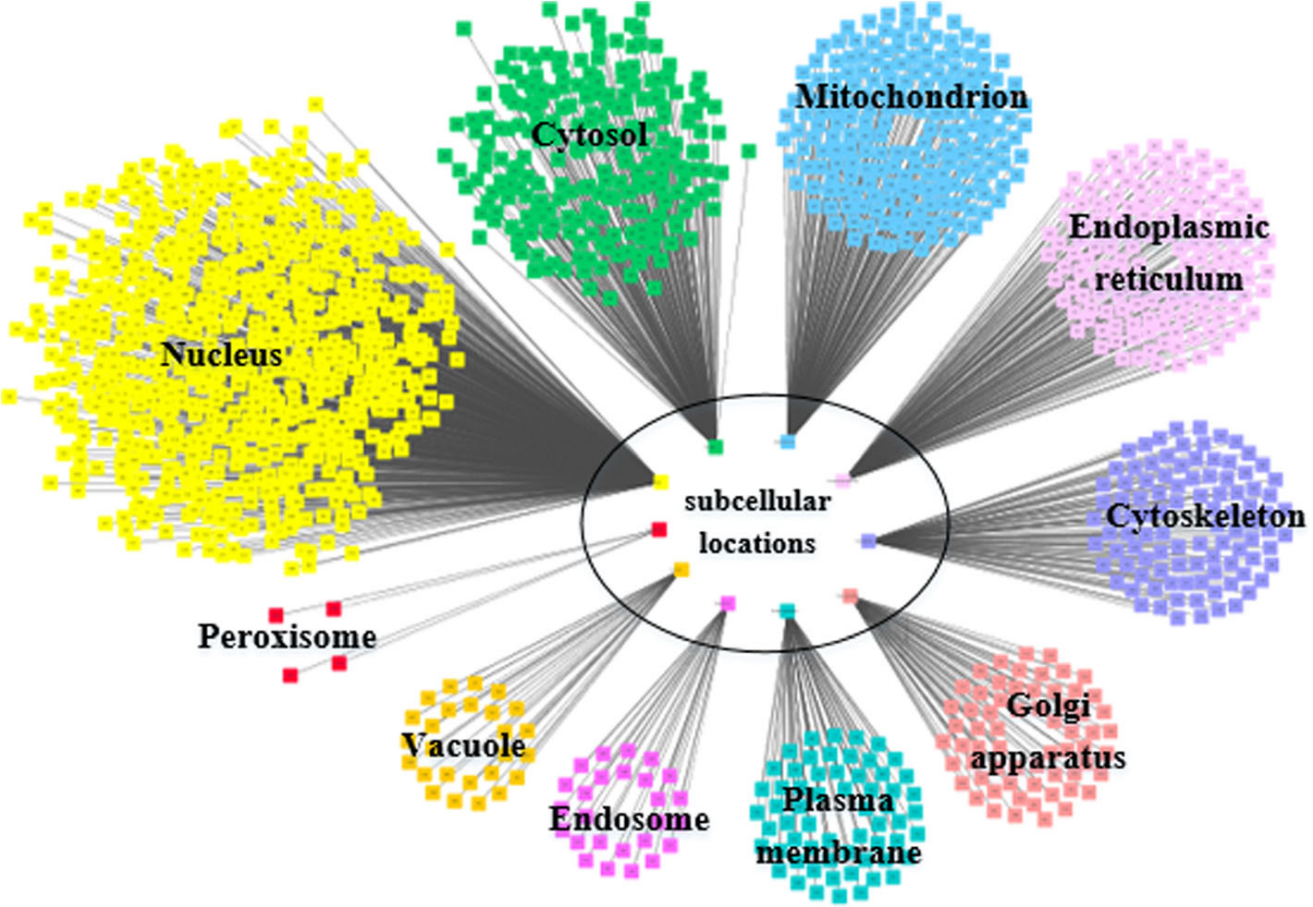
The relationship dataset. The nodes with different colors represent the distribution of the known essential proteins in different subcellular locations. There should have been 11 subcellular locations, but the known essential proteins only appear in the 10 subcellular locations other than the Extracellular space

In order to prove our hypothesis, we analyze the relationship between the final subcellular localization dataset *R* and the known essential protein dataset, the relationship dataset is defined as *S*, then each of the 11 subcellular locations is called *S*_*r*_, as shown in Fig. 2.

From Fig. 2 we can see that the known essential proteins appear most frequently in the Nucleus and it shows that the proteins in the Nucleus are more likely to be essential proteins. However, few of the known essential proteins appear in the Peroxisome, indicating that the proteins appear in the Peroxisome are essential proteins with a small probability.

If protein *i* exists in *R*, we calculate the frequency where each of the 11 subcellular locations appears, the corresponding score for each location is denoted as *F*_*i*_(*r*) by the following formula:

12 $$ {F}_i(r)=\left\{\begin{array}{c}\frac{S_r}{length(S)}, if\ i\cap R\\ {}\kern1em 0\kern1em , otherwise\end{array}\right. $$

where *length*(*S*) is the number of subcellular location records of the known essential proteins in the dataset *R*.

An efficient computational method is obtained to determine the subcellular localization score of a protein from the above-mentioned analysis. For this reason, we use the subcellular localization information to devise subcellular localization scores of proteins. For a given protein *i*, its subcellular localization score *SL*(*i*) is defined as the sum of scores of all the subcellular locations in which it appears.

13 $$ SL(i)=\sum \limits_{C(i)}{F}_i(r) $$

where *C*(*i*) denotes the set of corresponding subcellular locations in which protein *i* in the dataset *R.* Note that a protein may appear in multiple subcellular locations.

#### Protein complexes

Proteins often bind together to constitute protein complexes for carrying out their functions [33]. Based on the observation that essentiality is more likely to be the product of a protein complex rather than an individual protein [34] and proteins existed in complexes are tend to be essential compared to the proteins not appeared in complexes [26], in this subsection, we use two different protein complex datasets obtained from [35] that contain 270 and 425 complexes, respectively. After removing the repeated protein complexes, we collect 538 known protein complexes into a dataset, named *P*, denotes as *P* = {*P*_*1*_, *P*_*2*_, …, *P*_*k*_}.

A protein’s complex score is evaluated by the number of times it appears in the known protein complexes. For a given protein *i*, its protein complex score *PC*(*i*) is defined as follows:

14 $$ PC(i)=\sum \limits_{k=1}^M{T}_i(k) $$

15 $$ {T}_i(k)=\left\{\begin{array}{c}1,\kern1.25em if\ i\in {P}_k\\ {}\kern0.75em 0,\kern1em otherwise\end{array}\right. $$

where *M* is the number of the known protein complexes. If a protein exists in the known protein complexes, the value of its *PC* is the number of times it appears in the known protein complexes. If a protein does not appear in any protein complexes, the value of its *PC* is 0. We can clearly find that for a given protein *i*, it appears in more protein complexes and can get a higher value of *PC*.

### Pseudocode of FPE

Our proposed new algorithm FPE adopts the improved version of FPA algorithm by simulating the pollination process of flowers to identify essential proteins.

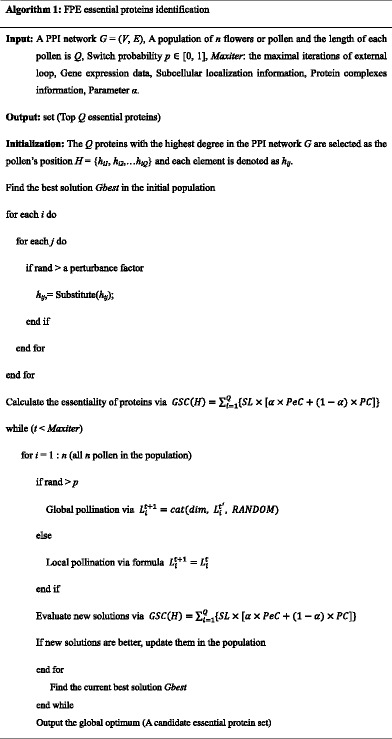



In FPE, first the *Q* proteins with the highest degree in the PPI network are selected as initial position of pollen to improve efficiency of FPE algorithm and using a perturbance factor that is a constant between [0, 1] to make sure that each pollen is different. Then, the measurement *GSC* is used to assess the quality of each candidate set. We redefine the update rules of the pollen’ s position and each pollen is updated by tailing the global optimal solution in each iteration since the global optimum can be viewed as a reliable guide for pollen to search better solution. The pseudo code of improved flower pollination algorithm for identifying essential proteins is described shown in Algorithm 1.

Switch probability *p* can be used to switch between global pollination and local pollination. The effect of *p* on the results will be discussed in experimental section and our experimental results demonstrate that the better result can be obtained when the value of *p* is 0.3.

## Results and discussion

In order to test whether our proposed algorithm FPE is effective for identifying essential proteins, we apply it to identify essential proteins of *S. cerevisiae*. First, we use the FPE algorithm to identify essential proteins and compare with ten other essential protein discovery methods: DC [11], SC [15], IC [17], EC [16], LAC [19], NC [18], PeC [22], WDC [32], UDoNC [23] and SON [25]. Then, the performance of FPE is evaluated in terms of the PR curve and the jackknife curve. After that, the modularity of proteins is used to confirm the performance of FPE. Finally, the effect of parameter *p* on the experimental results of proposed algorithm FPE is discussed.

### Experimental data

All the experiments in this study are based on the PPI network data of *S. cerevisiae* to identify essential proteins because it is the most complete data and has widely been used in the study of predicting essential proteins. The PPI network dataset of *S. cerevisiae* is downloaded from the DIP database [36]. The final yeast PPI network includes 5093 proteins and 24,743 interactions after the repeated interactions and the self-connecting interactions are removed. Other types of biological information used in this study are described as follows:

Gene expression data: The yeast gene expression data, GSE3431, are obtained from the Gene Expression Omnibus (GEO) database [37]. A total of 7074 gene products are used in our experiment.

Subcellular localization data: The protein subcellular localization dataset of *S. cerevisiae* is obtained from the subcellular localization database of COMPARTMENTS [38]. The yeast proteins have a total of 11 subcellular localizations as follows: Cytoskeleton, Golgi apparatus, Peroxisome, Cytosol, Endosome, Mitochondrion, Plasma membrane, Nucleus, Extracellular space, Vacuole, Endoplasmic reticulum. After preprocessing, it still includes 6892 subcellular localization records.

Protein complexes data: We integrate two real protein complex sets into one protein complex set. These two sets from [35] contain 270 and 425 complexes, respectively. The final known protein complex dataset contains 538 complexes, gathered from these two complex sets by removing the repeated protein complexes, named *P*.

Standard essential protein set: A list of the known essential proteins of *S. cerevisiae* is collected from the following databases: MIPS (Mammalian Protein-Protein Interaction Database) [39], SGD (Saccharomyces Genome Database) [40], DEG (Database of Essential Genes) [41], and SGDP (Saccharomyces Genome Deletion Project) [42]. There are 1285 essential proteins are collected in this dataset.

### Comparison of FPE with other essential protein discovery methods

To compare the performance of FPE with other previous essential protein discovery methods DC, SC, IC, EC, LAC, NC, PeC, WDC, UDoNC and SON, we first apply these ten methods on the yeast PPI network. Then, similar to most methods of predicting essential proteins, we rank all the proteins in descending order in the PPI network and select the top 100, top 200, top 300, top 400, top 500, and top 600 proteins as essential candidates. Finally, according to the standard essential protein dataset, the number of true essential proteins is detected by ten competing methods DC, SC, IC, EC, LAC, NC, PeC, WDC, UDoNC and SON in the yeast PPI network.

For our proposed FPE algorithm, first the global optimum, i.e., a candidate essential protein set is obtained based on the improved FPA, then we rank *Q* proteins in descending order from the obtained candidate set by using our redefined measurement *GSC* and select the top 100, top 200, top 300, top 400, top 500, and top 600 proteins as essential candidates, finally, we achieve the number of true essential proteins predicted by FPE.

**Fig. 3:**
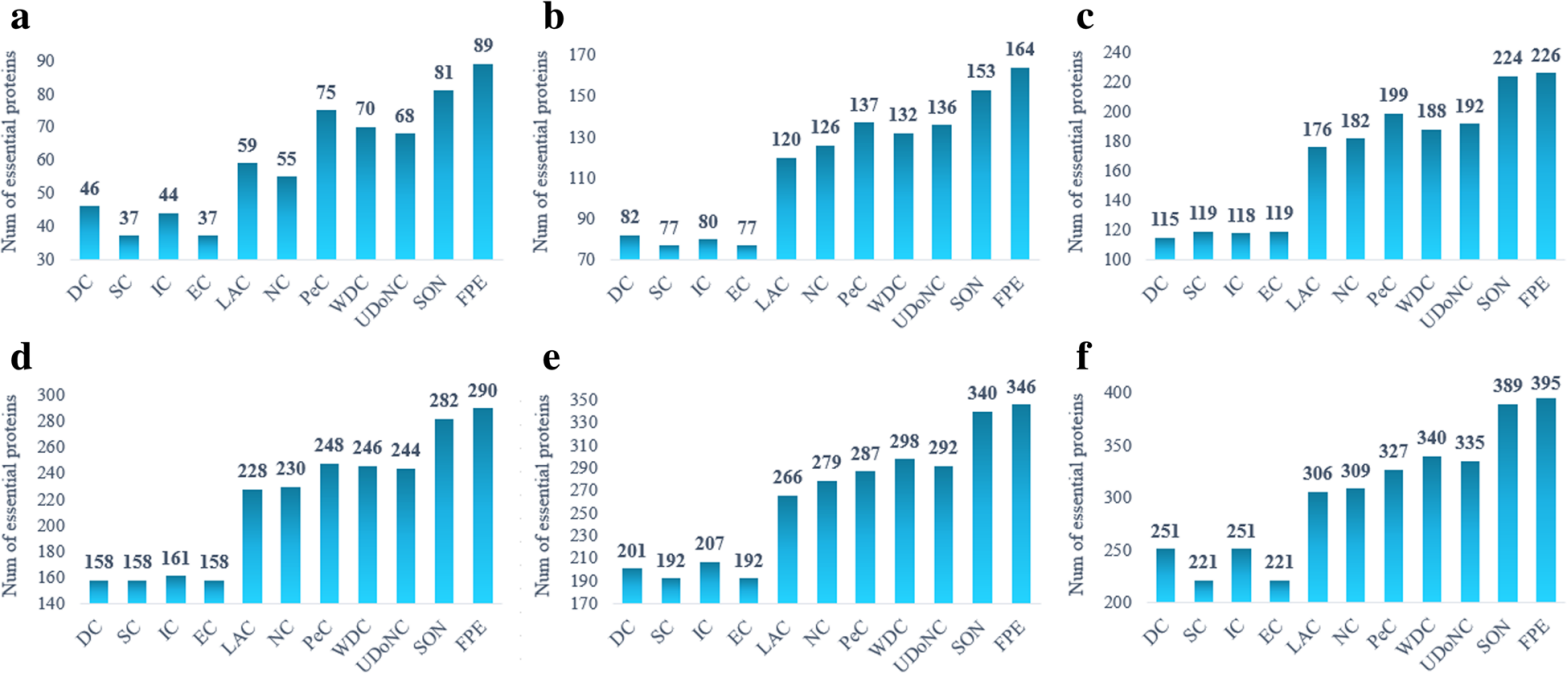
Comparison of FPE with other essential protein discovery methods. The ten competing methods are DC, SC, IC, EC, LAC, NC, PeC, WDC, UDoNC and SON. (**a**), (**b**), (**c**), (**d**), (**e**), and (**f**) show the results of these methods when select top 100, 200, 300, 400, 500, and 600 proteins as essential candidates, respectively. Note that the number of identification presented here is the result of FPE algorithm running ten times and then averaging the number of ten times

The comparison results are shown in Fig. 3. From Fig. 3 we can see that FPE has a better performance compared with the other ten essential protein discovery methods for predicting essential proteins from the yeast PPI networks. The number of true essential proteins identified by FPE is consistently higher than those generated by the ten previously proposed methods: DC, SC, IC, EC, LAC, NC, PeC, WDC, UDoNC and SON from top 100 to top 600 proteins. By choosing top 100 proteins, FPE can obtain a prediction precision of 89%. Especially compared to LAC, the improvements of FPE are 50.85, 36.67, 28.41, 27.19, 30.08 and 29.08% from top 100 to top 600 proteins, respectively.

It should be noted that the identification result of each time in our algorithm FPE with randomness due to the characteristics of the intelligent algorithm itself, but the result of each time is basically maintained within a stable range.

### Validation in terms of the precision-recall curve

**Fig. 4:**
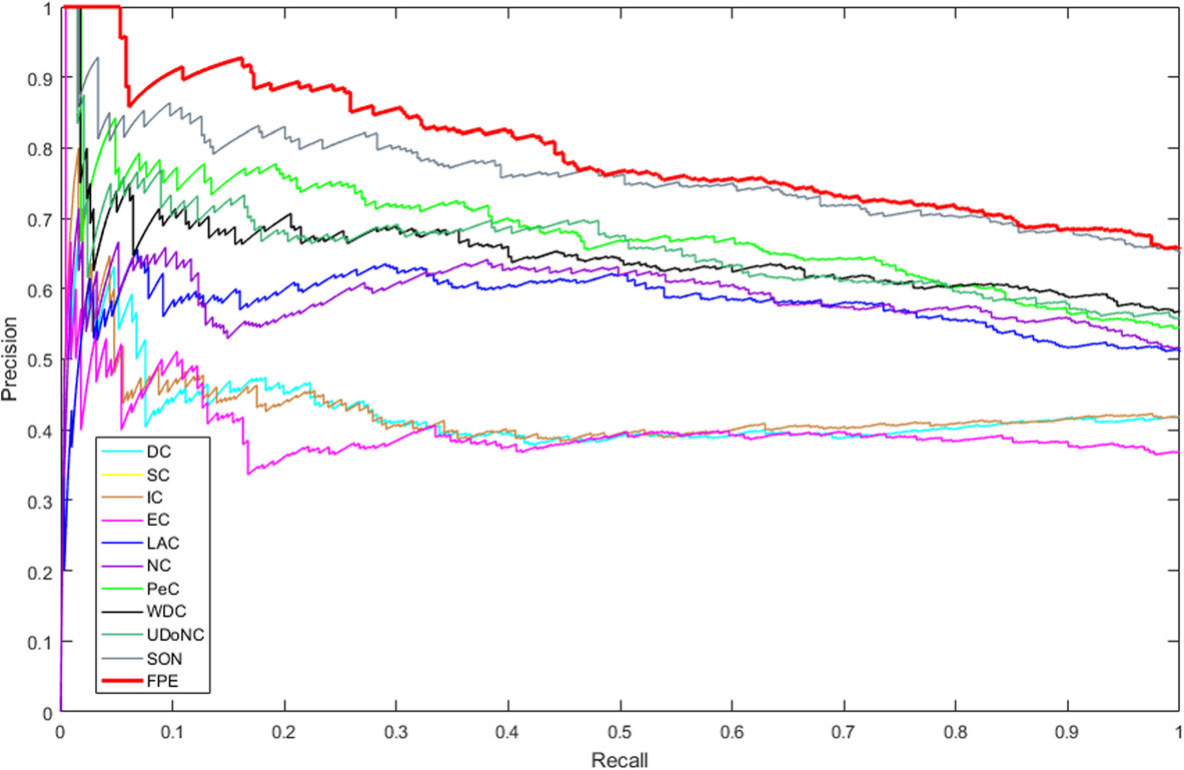
Validation in terms of the precision-recall curve. Comparison of DC, SC, IC, EC, LAC, NC, PeC, WDC, UDoNC, SON and FPE based on the validation of PR curve

In this subsection, we use precision-recall (PR) curve that is a common methodology to evaluate the performance of the proposed algorithm FPE. A comparison of FPE with the ten methods DC, SC, IC, EC, LAC, NC, PeC, WDC, UDoNC and SON for predicting essential proteins from the yeast PPI networks by using the PR curve is shown in Fig. 4.

From Fig. 4 we can see that the PR curve of FPE obtains the better result compared to the PR curves of ten other previously proposed essential protein discovery methods: DC, SC, IC, EC, LAC, NC, PeC, WDC, UDoNC and SON. The PR curves of EC and SC are almost the same.

We have known our algorithm FPE with randomness, but the result of each time remains in a stable range, the PR curve of the FPE algorithm here is randomly selecting from the ten times running results.

### Validation in terms of the jackknife curve

**Fig. 5:**
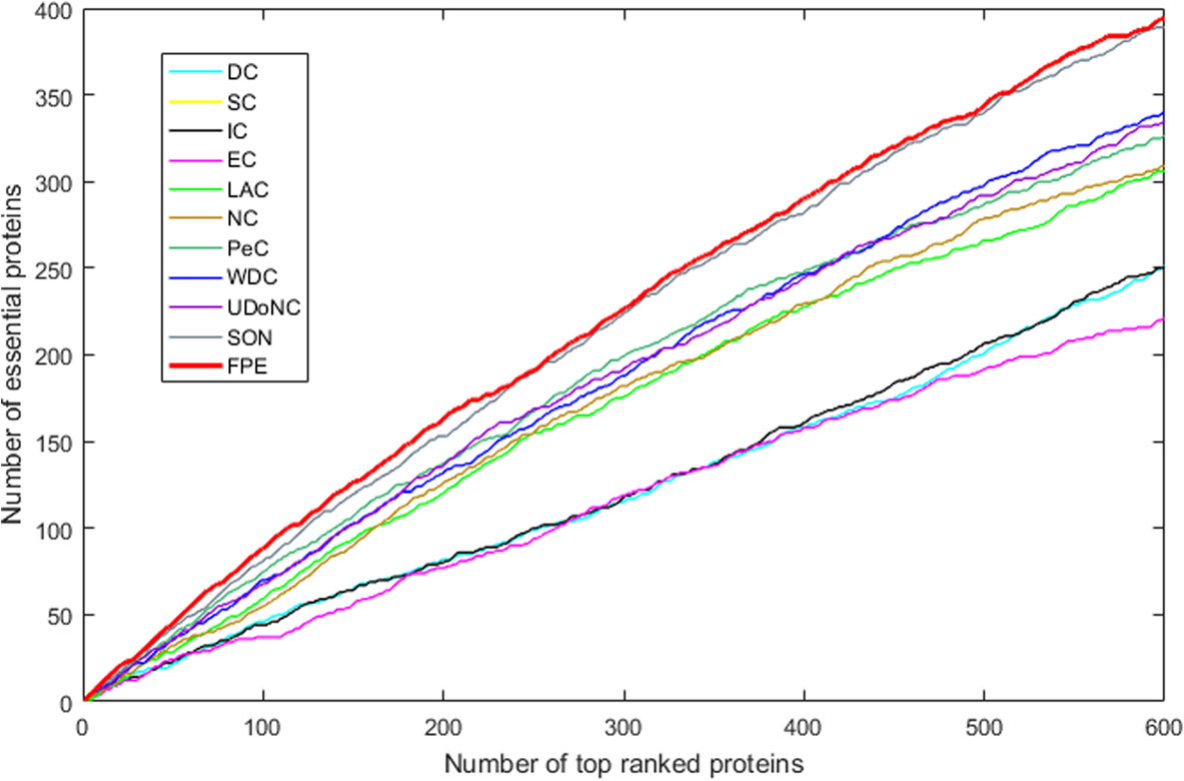
Validation in terms of the jackknife curve. Comparison of DC, SC, IC, EC, LAC, NC, PeC, WDC, UDoNC, SON and FPE based on the validation of jackknife method

To evaluate the effectiveness of FPE more generally, we further use the jackknife curve to illustrate the prediction results of DC, SC, IC, EC, LAC, NC, PeC, WDC, UDoNC, SON and our proposed algorithm FPE. The results are shown in Fig. 5, the x-axis represents the number of proteins are ranked by each essential protein discovery method and the y-axis is the cumulative count of true essential proteins.

The areas under the curves can measure the performances of the above-mentioned methods. As shown in Fig. 5, the jackknife curve of FPE is better than the other methods DC, SC, IC, EC, LAC, NC, PeC, WDC, UDoNC and SON for identifying essential proteins from the yeast PPI networks. It demonstrates that FPE is more effective than other ten methods for identifying essential proteins. The jackknife curves of EC and SC are almost the same. The jackknife curve uses the same running results of the FPE algorithm as the PR curve.

### Evaluation of the modularity of proteins predicted by FPE, DC and PeC

**Fig. 6:**
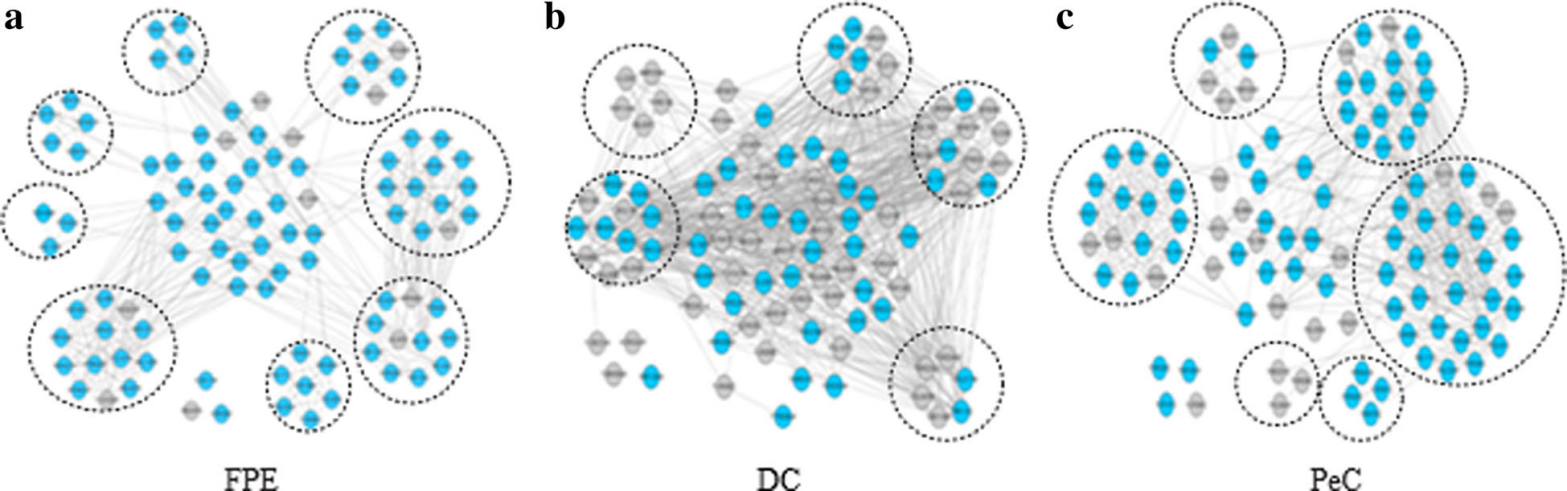
Evaluation of the modularity of proteins predicted by FPE, DC and PeC. Top 100 proteins in the yeast PPI networks identified by FPE, DC and PeC, respectively. The blue nodes represent the essential proteins and the grey nodes represent the non-essential proteins. In (**a**), MCODE discovers 8 functional modules. (**b**) shows that MCODE identifies 5 functional modules. (**c**) shows that MCODE detects 6 functional modules

Proteins often form protein complexes or functional modules to perform their biological functions. Therefore, we try to use protein modularity to assess the essential proteins predicted by FPE. To study the modularity of the proteins, we first choose the top 100 proteins identified by FPE, DC and PeC to construct three small PPI networks. Each small network consists of the top 100 proteins ranked by FPE, DC and PeC. Then, MCODE [43] is used to discover protein modules from the three small PPI networks. The results are shown in Fig. 6.

As shown in Fig. 6, the top 100 proteins ranked by FPE include 88 essential proteins (blue nodes in Fig. 6(a)), whereas DC only identifies 46. MCODE has detected eight modules in the PPI network of FPE, five modules in the PPI network of DC and six modules in the PPI network of PeC. From Fig. 6(b) and Fig. 6(c), there are modules that have not been discovered by MCODE. From the results, we can see that the essential proteins predicted by FPE show more obvious modularity than those identified by DC and PeC.

### Effects of the parameter *p*

In this subsection, we discuss the influence of the parameter *p* that is the switch probability on the prediction results of FPE. With *p* = 0, flowers do not perform the local pollination while with *p* = 1, flowers do not perform the global pollination. For this purpose, we set the switch probability *p* vary from 0.1 to 0.9. Then the FPE algorithm is ran ten times from *p* = 0.1 to *p* = 0.9, respectively, and we calculate their average. Finally, the number of true essential proteins identified by FPE is shown in Table 3.

**Table 3:** The number of true essential proteins identified by FPE with different *p*

*p*	Top 100	Top 200	Top 300	Top 400	Top 500	Top 600
0.1	**89**	163	225	287	344	393
0.2	**89**	163	225	289	343	393
0.3	**89**	**164**	226	290	**346**	**395**
0.4	**89**	**164**	225	289	343	390
0.5	**89**	163	227	289	344	391
0.6	88	163	226	288	342	387
0.7	88	163	226	288	342	385
0.8	88	163	227	**291**	343	384
0.9	88	163	**228**	289	343	384

The data in boldface represents the maximum value in each column

According to Table 3, we can see that the differences between the results of *p* < 0.6 and 0.6 ≤ *p* < 1.0 are obvious, when *p* < 0.6, the number of true essential proteins identified by FPE is almost higher than 0.6 ≤ *p* < 1.0, which implies that selecting *p* < 0.6 is a good choice. Moreover, when *p* = 0.3, more superior results can be obtained and further demonstrate that setting the value of switch probability *p* to be 0.3 is the best choice for predicting essential proteins of FPE. Hence, in this study, we determine the optimal value to be *p* = 0.3.

## Conclusions

The identification of essential proteins is very significant to understand the minimal requirements for cellular life and disease study. In this study, we propose a new algorithm FPE based on the improved flower pollination algorithm to identify essential proteins by integrating gene expression data, subcellular localization and protein complexes information with the topological properties of PPI networks.

To test whether the proposed algorithm is effective, we apply our proposed algorithm FPE on the PPI network of *S. cerevisiae*. First, the comparisons of FPE with ten previous proposed methods DC, SC, IC, EC, LAC, NC, PeC, WDC, UDoNC and SON have been made in terms of the number of predicted true essential proteins, as well as the PR curve and the jackknife curve. Then, we further analyze the modularity of proteins and the effect of the switch probability *p* on the identification results. Both the numerical and the graphical experiment results show that FPE is more competitive than other methods for the identification of essential proteins.
